# Molecular Cloning and mRNA Expression of Heat Shock Protein Genes and Their Response to Cadmium Stress in the Grasshopper *Oxya chinensis*

**DOI:** 10.1371/journal.pone.0131244

**Published:** 2015-07-02

**Authors:** Yuping Zhang, Yaoming Liu, Jianzhen Zhang, Yaping Guo, Enbo Ma

**Affiliations:** 1 Institute of Applied Biology, Shanxi University, Taiyuan, Shanxi, China; 2 Biology Department, Taiyuan Normal University, Taiyuan, Shanxi, China; 3 College of Life Science, Shanxi University, Taiyuan, Shanxi, China; University of Palermo, ITALY

## Abstract

Heat shock proteins (*Hsps*) are highly conserved molecular chaperones that are synthesized in response to stress. In this study, we cloned the full-length sequences of the *Grp78* (glucose-regulated protein 78), *Hsp70*, *Hsp90*, and *Hsp40* genes from the Chinese rice grasshopper *Oxya chinensis*. The full-length cDNA sequences of *OcGrp78*, *OcHsp70*, *OcHsp90*, and *OcHsp40* contain open reading frames of 1947, 1920, 2172, and 1042 bp that encode proteins of 649, 640, 724, and 347 amino acids, respectively. Fluorescent real-time quantitative PCR (RT-qPCR) was performed to quantify the relative transcript levels of these *Hsp* genes in different tissues and developmental stages. The mRNAs encoding these four *Hsp* genes were present at all developmental stages and in all tissues examined but were expressed at varying levels. Additionally, we investigated the mRNA expression profiles of these four *Hsps* in *O*. *chinensis* subjected to Cadmium (Cd) stress. *OcGrp78*, *OcHsp70*, *OcHsp90*, and *OcHsp40* mRNA expression was induced under acute Cd stress; the levels reached a maximum within a short time (6 h), were reduced significantly at 12 h, and were lowered to or below control levels by 48 h. Regarding induction efficiency, *OcHsp70* was the most sensitive gene to acute Cd stress. Chronic Cd exposure showed that dietary Cd treatment induced increased *OcGrp78*, *OcHsp90*, and *OcHsp40* expression. However, dietary Cd induced a significant reduction of *OcHsp70* expression. In the period tested, no significant difference in the mortality of the grasshoppers was observed. Our results suggest that these four *Hsps* genes, especially *OcHsp70*, are sensitive to acute Cd stress and could be used as molecular markers for toxicology studies. However, our results also indicate that *OcHsp70* is not suitable for use as a molecular marker of chronic Cd contamination.

## Introduction

Heat shock proteins (Hsps), or stress proteins, are a group of conserved proteins that are synthesized by organisms upon exposure to environmental stressors, including heat shock, radiation, pesticides, metal, and other environmental contaminants. As molecular chaperones, Hsps play an essential role by assisting in the correct folding of proteins to maintain cellular homoeostasis under stress conditions [1–2]. Hsps can be categorized into five families, which are named according to their molecular weights, conserved sequences, and molecular functions: Hsp100, Hsp90, Hsp70, Hsp60, Hsp40 and the small Hsps [3]. Eukaryotes have multiple Hsp70 family members that are located in the lumen of the endoplasmic reticulum (e.g., glucose-regulated protein 78, Grp78) and in the cytoplasm, including constitutive heat shock cognate 70 protein (Hsc70) and inducible Hsp70 [4]. *Hsp* gene expression is widely accepted as a suitable molecular indicator of adverse biological effects because Hsps respond to minor environmental stress and are evolutionarily conserved [5–6]. Therefore, much of the research on cellular responses to metals and other pollutants has focused on Hsps, especially Hsp70 because of its high conservation and sensitivity to stress factors [6–10].

Cadmium (Cd) is produced as a result of industrial and agricultural processes and is a highly toxic metal to many organisms; therefore, recent research has focused on the toxicology of and the molecular response mechanism to Cd. Cd is best known as a widespread pollutant that can have harmful effects on human health. Cd accumulates through the food chain or respiratory system due to both natural and factitious release into the environment. Metal accumulation can affect many physical and biochemical processes in living organisms, including the effects of Hsp responses to metal [11–13].

*Oxya chinensis* (Orthoptera: Acridoidea) is widely distributed in China and is an important agricultural rice pest. Metals in the agricultural environment can transfer into the bodies of grasshoppers through the food chain. Our previous studies have shown that Cd accumulates in grasshoppers, inducing antioxidant enzyme activity and metallothionein (MT) expression to resist its toxic effects [14–17]. However, studies on the effects of Cd in grasshoppers at the molecular level of Hsps have not been reported.

The present study aimed to clone and identify four full-length cDNAs of *Hsp* genes (*Hsp70*, *Grp78*, *Hsp90*, and *Hsp40*) from *O*. *chinensis*. We then analyzed the expression patterns of these four *Hsp* genes in different tissues and at different developmental stages. We also investigated the temporal expression profile of *Hsp* mRNAs in *O*. *chinensis* following Cd injection. Finally, we analyzed the mRNA expression patterns of *Hsp* genes in *O*. *chinensis* fed Cd-infected wheat seedlings.

## Materials and Methods

### Insect and tissue collection

*O*. *chinensis* eggs were collected from the area surrounding Jinyang Lake, Shanxi Province, China in October 2011. The eggs were incubated in a growth chamber (MGC-350NR2, Shanghai, China) at 26±1°C and 60% relative humidity (RH) with a 14:10-h light:dark (L:D) photoperiod. After hatching, insects were reared on fresh wheat seedlings. Tissue samples (brain, epidermis, foregut, midgut, gastric caeca, hindgut, Malpighian tubule, and fat body) were collected from 5th instar nymphs. Eggs that had not started hatching and whole bodies of 1st-5th instar nymphs and adults were collected to analyze the stage-specific distribution of *Hsp* genes at day 3 of each stage. We explicitly confirmed that no specific permissions were required for these locations and activities and that the field studies did not involve endangered or protected species.

### RNA isolation, cDNA synthesis, and degenerate PCR

Total RNA was extracted from the samples using TRIzol (Invitrogen, USA) according to the manufacturer’s instructions. cDNA was synthesized by reverse transcription in 25 μL reactions containing 1 μg of total RNA, 40U RNase inhibitor, 1 μL dNTP mixture (10 mM), 1 μL Oligo d(T)_18_ primer (500 ng μL^-1^), and PrimeScript reverse transcriptase (TaKaRa, Japan) at 42°C for 1 h. The reaction mixture was stored at -20°C.

Primers were designed using Primer Premier 5.0 based on published sequences in other insects (Table 1). Degenerate PCR was conducted using Taq DNA Polymerase (Tiangen, Beijing, China) with a reaction volume of 25 μL. Fragments were purified using a Gel Extraction Mini Kit (Tiangen, Beijing, China), cloned into the pGEM-T Easy vector (Promega, Madison, WI, USA) and then sequenced.

**Table 1:** List of primers used in RT-PCR, RACE-PCR, and real time PCR.

Gene	Primer Name	Function	Primer sequence
*Hsp70*	Hsp70-F	Fragment cloning	GARATHATHGCNAAYGA
	Hsp70-R	Fragment cloning	ACRTCRAANGTNCCNCC
	Hsp70-5′	5′-RACE PCR	GTAATAACTGCCTCACGAACCTGCCCAC
	Hsp70-3′	3′-RACE PCR	GGTGCTGCGGATTATCAACGAGCCAACT
	Hsp70-FC-F	Full length cloning	CAGAAGAAAATGCCGAAGATTCC
	Hsp70-FC-R	Full length cloning	TTAACAGTTCACTTAGTCAACCTCCTC
	Hsp70-RT-F	Real time PCR	AGCACTGACCGATGCCAAGAT
	Hsp70-RT-R	Real time PCR	GCACTGACCGATGCCAAGAT
*Grp78*	Grp78-F	Fragment cloning	GARATHATHGCNAAYGA
	Grp78-R	Fragment cloning	ACRTCRAANGTNCCNCC
	Grp78-5′	5′-RACE PCR	ATACGACGGCGTGATTCTGTTTCCTTG
	Grp78-3′	3′-RACE PCR	CCTACGGACTGGACAAGAAAGAGGGAGA
	Grp78-FC-F	Full length cloning	ATAAACCCACCATGAGGGGAATTC
	Grp78-FC-R	Full length cloning	GACCGACTTTTACAGTTCATCCTT
	Grp78-RT-F	Real time PCR	CCAAAGGTGCAGCAACTTGTAAA
	Grp78-RT-R	Real time PCR	CAATCGCATCTGTATCCTGTTCT
*Hsp40*	Hsp40-F	Fragment cloning	AARTAYCAYCCNGAYAARAAY
	Hsp40-R	Fragment cloning	GCYTT CCA NCCNGG YTT NAC
	Hsp40-5′	5′-RACE PCR	GTAAATGGGTCATCCAACTCC
	Hsp40-3′	3′-RACE PCR	AGTTGGATGACCCATTTACCT
	Hsp40-FC-F	Full length cloning	ATCGTTGAAGATGGGGAAAG
	Hsp40-FC-R	Full length cloning	CCCTCACAGTAACATTCAAT
	Hsp40-RT-F	Real time PCR	AGTTGGATGACCCATTTACCT
	Hsp40-RT-R	Real time PCR	TTTCTTCGTGCAACCTCTTAG
*Hsp90*	Hsp90-F	Fragment cloning	ACNAARGCNTTYATGGARGC
	Hsp90-R	Fragment cloning	TCRCARTTRTCCATDATRAA
	Hsp90-5′	5′-RACE PCR	CAGATTGGCTTGGTCTTGTTT
	Hsp90-3′	3′-RACE PCR	CCGAAGATTGAAGATGTTGGA
	Hsp90-FC-F	Full length cloning	TGTCCAAGATGCCGGAGGAC
	Hsp90-FC-R	Full length cloning	TTAATCAACTTCCTCCATTCGAGAG
	Hsp90-RT-F	Real time PCR	AAACAAGACCAAGCCAATCTG
	Hsp90-RT-R	Real time PCR	AATACCCTGCGGACATACAAT
*β-actin*	*β*-actin-RT-F	Real time PCR	CGAAGCACAGTCAAAGAGAGGTA
	*β*-actin-RT-R	Real time PCR	GCTTCAGTCAAGAGAACAGGATG

### Rapid amplification of cDNA ends (RACE)

cDNA was synthesized using the SMART RACE cDNA amplification kit (Clontech, USA) according to the manufacturer’s instruction. For amplification of the 3' and 5' end cDNA sequences, the SMART RACE cDNA amplification kit (Clontech) was used according to the manufacturer’s protocol. Amplified products from each reaction were purified using a Gel Mini purification kit (Tiangen), and the isolated amplification products were quantified, subcloned into the pGEM-T Easy vector (Promega) and then sequenced.

### Sequence characterization and alignment

Open reading frame (ORF) prediction and cDNA translation into amino acid sequences were performed using the translation tool in ExPaSy (http://www.expasy.org/tools/dna.html). The molecular mass and isoelectric points (pIs) were predicted based on the amino acid sequences. The deduced amino acid sequences of the Hsp genes in *O*. *chinensis* (*OcHsps*) were compared with those Hsp sequences in other insects deposited in GenBank using the ‘BLAST-N’ tools available on the National Center for Biotechnology Information (NCBI) website and GENEDOC software (Nicholas et al., 1997). A phylogenetic tree was constructed by MEGA 5.0 using the neighbor-joining method.

### Cd treatment

#### Acute Cd treatment

Healthy and uniform 5th instar nymphs were selected for acute Cd treatment. To examine the induction profile of *OcHsp*s at different time points, the nymphs were exposed to Cd for 48 h and sampled at set intervals. A Cd concentration of 2.7 mg·kg^-1^ was used to avoid increased mortality with prolonged treatment. To examine the induction profile of *OcHsp*s at different Cd concentrations, 5th instar nymphs were exposed to different doses of Cd and sampled after 6 h. The concentrations used were 2.7, 5.4, and 10.8 mg·kg^-1^; these concentrations can cause approximately 5–10% mortality according to the previously determined LC_5_ (2.49 mg·kg^-1^) and LC_10_ (11.52 mg·kg^-1^) values at 48 h. Then, 4 μL of Cd solution at the various concentrations was injected into the hemocoel through the membrane between the second and third abdominal segments using a micro syringe. Distilled water was used as a control. Ten insects (5 females and 5 males) were used for gene expression analysis at 0 h, 2 h, 6 h, 12 h, 24 h, and 48 h after injection.

#### Chronic Cd treatment

In this experiment, 1st instar nymphs were fed on wheat seedlings cultured with a CdCl_2_ solution. The Cd^2+^ concentration in the solution was 20, 40, or 80 mg L^-1^, and distilled water was used as a control. The Cd^2+^ concentrations were selected according to a previous study [16]. The Cd concentrations in the wheat seedlings were 0, 2.6±0.3, 6.3±0.8, and 13.4±1.5 mg·kg^-1^ dry weight with distilled water and 20, 40, and 80 mg L^-1^ CdCl_2_ solutions, respectively. Samples were collected from 3-day-old adults. Ten individual adults (5 females and 5 males) were collected per replicate. The samples were immediately frozen in liquid nitrogen and stored at -80°C until used for RNA extraction.

### Quantitative real time PCR (qPCR)

A qPCR assay was carried out to examine the *Hsp* gene mRNA levels; the primers used are shown in Table 1. qPCR was performed using an ABI 7300 real-time PCR detection system (ABI, USA) and Maxima SYBR Green qPCR Master Mix kit (TaKaRa, Dalian, China). The relative *Hsp* gene mRNA levels were assessed using the following cycling parameters: an initial denaturation at 95°C for 3 min followed by 40 cycles of 95°C for 20 s, 55°C for 20 s and 72°C for 20 s. After PCR, the homogeneity of the PCR product was confirmed by melting curve analysis. *β*-actin was used as an internal control, and the ratios of *Hsp*s/*β*-actin mRNA expression were calculated. The fold changes in all experiments were calculated according to the 2^**-**ΔΔCt^ method [18]. qPCR was repeated three times for each gene. Each replication comprised two technical replicates.

### Statistical analysis

Statistical analyses were carried out using SPSS 16.0 software (SPSS Inc., Chicago, IL, USA). The SPSS program was used for the statistical analysis of differences in mRNA expression by analysis of variance (ANOVA). The differences among means were determined using Duncan’s multiple comparison, and the significance level was set at *P* < 0.05.

## Results

### Analysis of cDNAs and the deduced amino acid sequences of *OcHsps*

The cDNA and protein characteristics of four *Hsp* genes are shown in Table 2. Classical HSP protein signature motifs, including IDLGTYYS (aa 9–16), IFDLGGGTFDVSIL (aa 197–210), and IVLVDDSTRIPKIQA (aa 335–349), were found in *OcHsp70*. The ATP/GTP-binding site AEAFLGGQ (aa 131–138), the non-organellar consensus motif RARFEEL (aa 300–306), and the conserved cytosolic EEVD motif in the C-terminus were also observed in *OcHsp70* (S1 Fig). *OcGrp78* also has three signature sequences commonly found in eukaryotic Hsps, including IDLGTTYS (aa 31–38), VFDLGGGTFDVSL (aa 219–231), and IVLVGGSTRIPKVQQ (aa 356–370). The endoplasmic HSP70-specific motif (KDEL) is located at the C-terminus. In addition, a special ATP/GTP binding site, AEAYLGKP (aa 154–161), was found in *OcGrp78* (S2 Fig). *OcHsp90* contains a conserved MEEVD motif and five characteristic protein sequences of the Hsp90 family, NKEIFLRELISNSSDALDKIR (aa 36–56), LGTIAKSGT (aa 103–111), IGQFGVGFYSAYLVAD (aa 127–142), LKLYVRRVFI (aa 353–362), and GVVDSEDLPLNISRG (aa 379–393) (S3 Fig). *OcHsp40* has three distinct regions. The first region is the J-domain (the N-terminal 73 amino acids), which is the most conserved region in these proteins. The second domain (aa 81–125) is referred to as the G/F domain and is rich in glycine and phenylalanine. The third motif is located at the C-terminal end and includes the substrate binding sites of Hsp40 (S4 Fig). The full-length nucleotide sequences and the deduced amino acid sequences are shown in the supporting information, and the characteristic sequences described above are indicated in the figures.

**Table 2:** Characteristics of the four *Hsps* in *O*. *chinensis*.

Gene names	GenBank accession No.	Full length (bp)	ORF (bp)	Amino acids (aa)	Predicted molecular weight (kDa)	Isoelectric point (pI)
*OcHsp70*	JQ859844	2340	1920	640	70	5.94
*OcGrp78*	JQ859843	2360	1947	649	72	5.79
*OcHsp90*	JQ859845	2442	2172	724	83	4.99
*OcHsp40*	JQ859846	1749	1041	347	36	9.28

### Phylogenetic relationship of *OcHsps* with those in other insects

**Fig 1:**
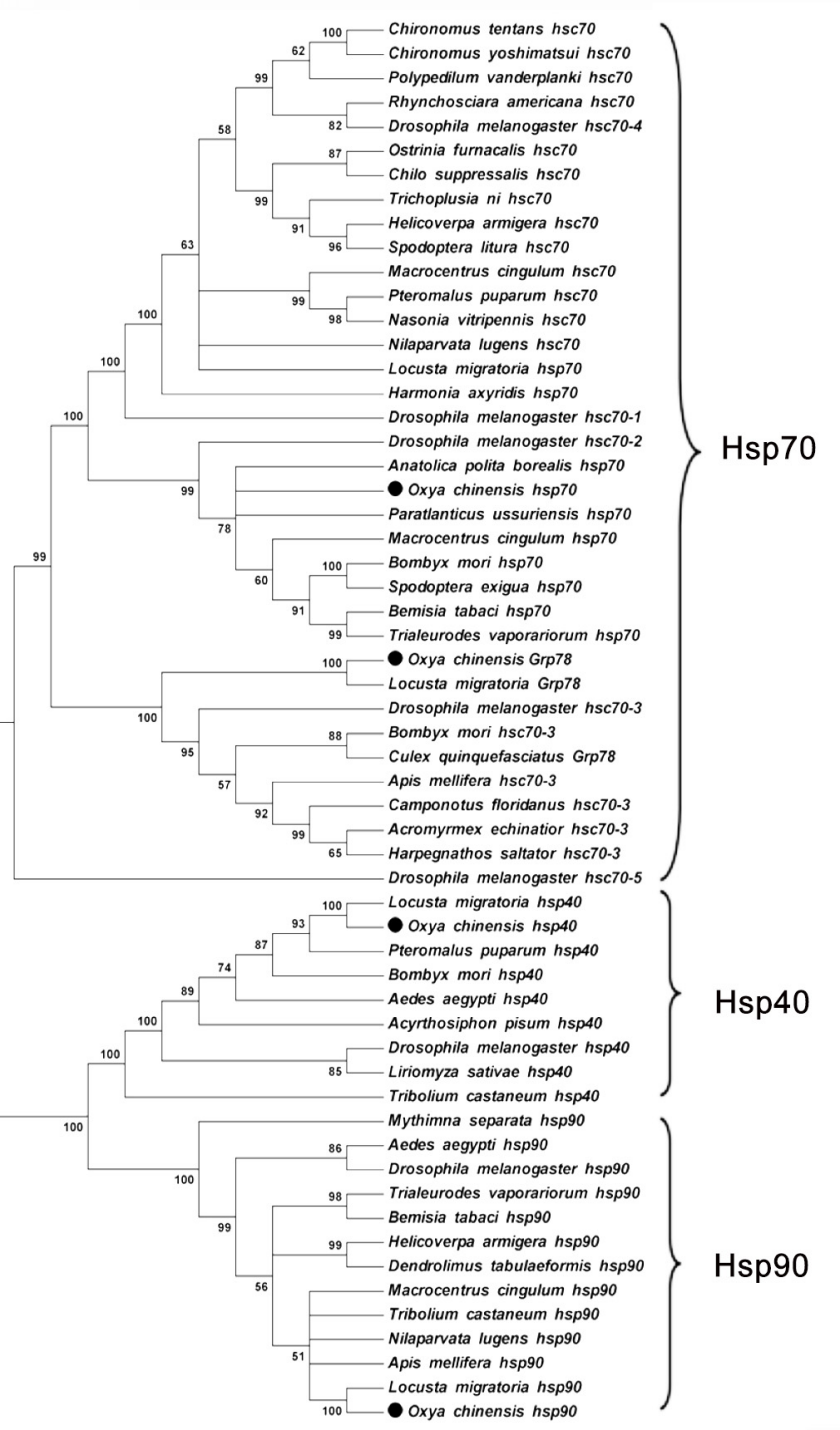
Phylogenetic analysis of *O. chinensis Hsps* and *Hsps* from other insects. Multiple alignment of the sequences used in the analysis was conducted using CLUSTAL W. A neighbor-joining tree construction program (MEGA 5.0) was used for this phylogenetic analysis. Information on the sequences used in this analysis is shown in Table 3.

To investigate the relationship of *Hsp* genes in *O*. *chinensis* with those in other insects, a phylogenetic analysis of their deduced protein sequences was performed using the neighbor-joining method and based on their sequences in other insects obtained from GenBank. Fig 1 shows that *OcGrp78*, *OcHsp70*, *OcHsp90*, and *OcHsp40* genes belong to the Hsp70, Hsp90, and Hsp40 families, respectively. The amino acid sequences of *OcHsp70*, *OcHsp90*, and *OcHsp40* are closest to the sequences obtained for Orthoptera insects. The amino acid sequence of *OcGrp78* was closest to that of *Grp78* in *Locust migratoria*. Our analysis also placed the *Grp78* sequence in a group containing *Hsc70-3* from other insects.

**Table 3:** A complete list of species used in the phylogenetic analysis and accession numbers for Hsp sequences.

Gene name in the Phylogenetic tree	Accession Number	Gene name in the Phylogenetic tree	Accession Number
*Chironomus tentans Hsc70*	AAN14525	*Anatolica polita borealis Hsp70*	ABQ39970
*Chironomus yoshimatsui Hsc70*	AAN14526	*Harmonia axyridis Hsp70*	ABR92405
*Polypedilum vanderplanki Hsc70*	ADM13381	*Macrocentrus cingulum Hsp70*	ACD84944
*Rhynchosciara americana Hsc70*	ABD63902	*Bombyx mori Hsp70*	BAF69068
*Trichoplusia ni Hsc70*	AAB06239	*Spodoptera exigua Hsp70*	ACN78407
*Ostrinia furnacalis Hsc70*	AEO19923	*Bemisia tabaci Hsp70*	ADO14473
*Chilo suppressalis Hsc70*	BAE44308	*Trialeurodes vaporariorum Hsp70*	ACH85201
*Helicoverpa armigera Hsc70*	ACL31668	*Nilaparvata lugens Hsp90*	ADE34169
*Spodoptera litura Hsc70*	ADK55518	*Trialeurodes vaporariorum Hsp90*	ACH85202
*Nilaparvata lugens Hsc70*	ADE34170	*Bemisia tabaci Hsp90*	ADO14474
*Pteromalus puparum Hsc70*	ACA53150	*Aedes aegypti Hsp90*	EAT36187
*Macrocentrus cingulum Hsc70*	ACD84943	*Macrocentrus cingulum Hsp90*	ACE77780.
*Nasonia vitripennis Hsc70*	NP_001166228	*Apis mellifera Hsp90*	NP_001153536
*Drosophila melanogaster Hsc70-1*	AAN11820	*Locusta migratoria Hsp90*	AAS45246
*Drosophila melanogaster Hsc70-2*	AAF54899	*Tribolium castaneum Hsp90*	ABR32189
*Drosophila melanogaster Hsc70-3*	AAN09301	*Helicoverpa armigera Hsp90*	ADM26743
*Drosophila melanogaster Hsc70-4*	AAO41568	*Mythimna separata Hsp90*	ABY55234
*Drosophila melanogaster Hsc70-5*	AAF58270	*Dendrolimus tabulaeformis Hsp90*	ABM89111
*Bombyx mori Hsc70-3*	AEI58998	*Drosophila melanogaster Hsp90*	NP_523899
*Apis mellifera Hsc70-3*	NP_001153524	*Bombyx mori Hsp40*	BAD90846
*Camponotus floridanus Hsc70-3*	EFN61604	*Drosophila melanogaster Hsp40*	NP_523936
*Acromyrmex echinatior Hsc70-3*	EGI70210	*Liriomyza sativae Hsp40*	ABE57132
*Harpegnathos saltator Hsc70-3*	EFN86831	*Pteromalus puparum Hsp40*	ACR44221
*Locusta migratoria Grp78*	ACS75352	*Locusta migratoria Hsp40*	ABC84495
*Culex quinquefasciatus Grp78*	XP_001845218	*Tribolium castaneum Hsp40*	XP_969979
*Paratlanticus ussuriensis Hsp70*	AEP68850	*Aedes aegypti Hsp40*	EAT45096
*Locusta migratoria Hsp70*	AAP57537	*Acyrthosiphon pisum Hsp40*	NP_001156836
***Oxya chinensis Hsp70***	**JQ859844**	***Oxya chinensis Hsp40***	**JQ859846**
***Oxya chinensis Grp78***	**JQ859843**	***Oxya chinensis Hsp90***	**JQ859845**

### mRNA expression of *OcHsps* in tissues not challenged with Cd

**Fig 2:**
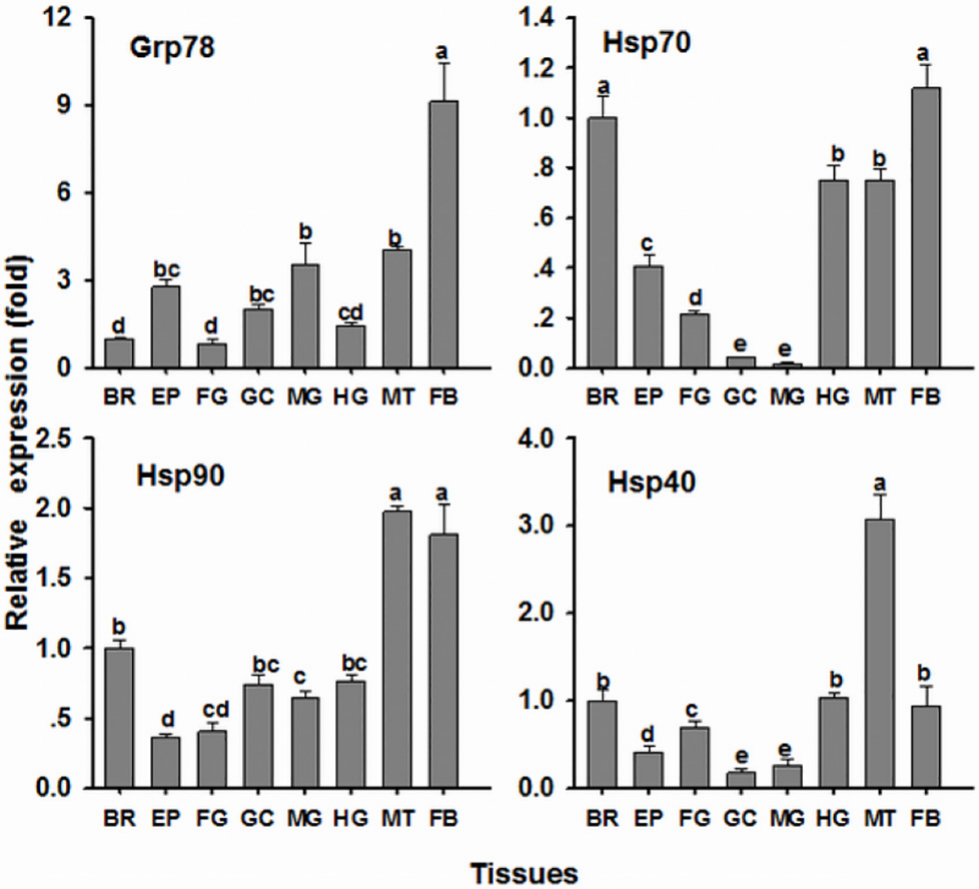
The tissue expression pattern of *O. chinensis Hsp*s in 5th instar nymphs. The bars represent the mean ± SE (n = 3) of target gene mRNA expression in different tissues. The small letters on the bars indicate significant differences between tissues (Duncan’s multiple comparison, *P*< 0.05). BR: brain; EP: epidermis; FG: foregut; GC: gastric caecum; MG: midgut; HG: hindgut; MT: Malpighian tubule; FB: fat body. The mRNA level in the brain was arbitrarily taken as 1.0.

*OcGrp78*, *OcHsp70*, *OcHsp90*, *and OcHsp40* mRNA expression was examined in several different tissues (Fig 2). ANOVA showed that *OcGrp78*, *OcHsp70*, *OcHsp90*, *and OcHsp40* mRNA expression is significantly different in different tissues (*OcHsp70*, *P*<0.001; *OcGrp78*, *P* = 0.001; *OcHsp90*, *P*<0.001; *OcHsp40*, *P*<0.001). *OcGrp78* expression was highest in the fat body, followed by the Malpighian tubule and midgut, which was 9.13-, 4.1-, and 3.5-fold higher than the brain, respectively. Relatively high *OcHsp70* expression was observed in the fat body and brain, followed by the Malpighian tubule and hindgut, whereas the lowest expression was found in the gastric caecum and midgut, which was only 4.3% and 1.9% of the brain expression, respectively. *OcHsp90* expression was relatively high in the Malpighian tubule and fat body, was lower in the brain, gastric caecum, hindgut, and midgut, and was lowest in the foregut and epidermis. The order of relative *OcHsp40* expression was the Malpighian tubule, followed by the fat body, hindgut, and brain, and then by the foregut, epidermis, gastric caecum and hindgut.

### *OcHsps* mRNA expression at developmental stages when not challenged with Cd

**Fig 3:**
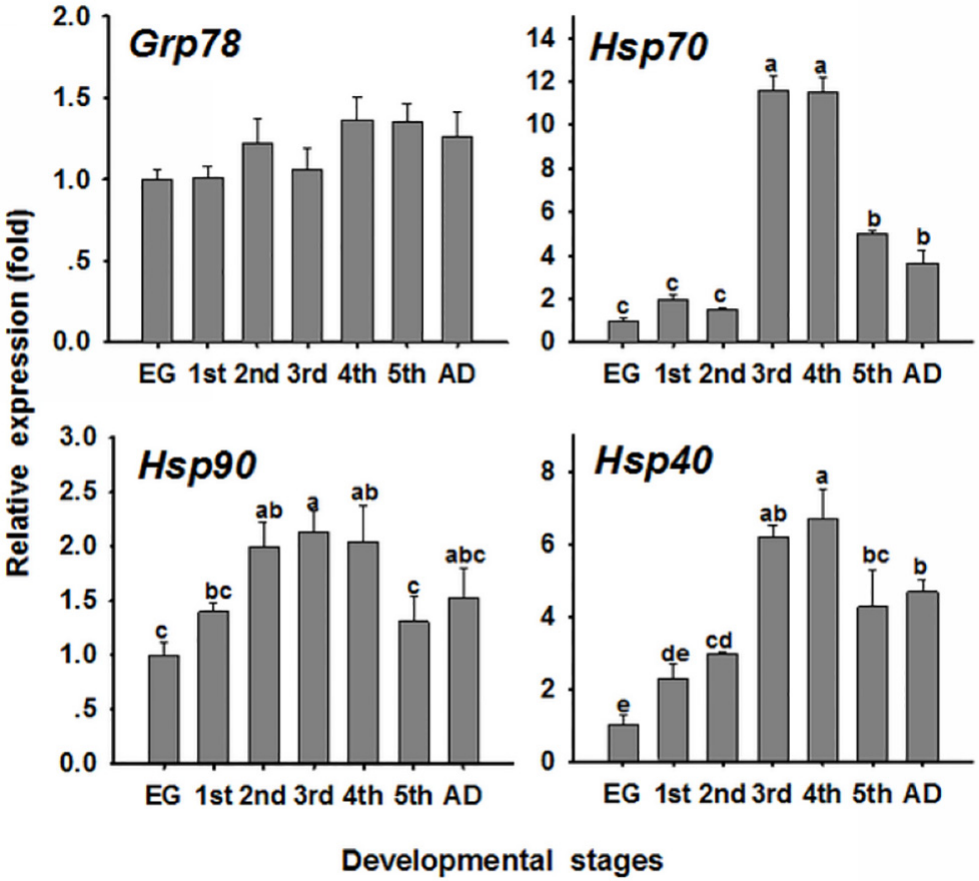
The developmental expression pattern of *O. chinensis Hsp*s in eggs, nymphs and adults. The bars represent the mean ± SE (n = 3) of target gene mRNA expression at different developmental stages. The different small letters on the bars indicate significant differences between developmental stages (Duncan’s multiple comparison, *P*< 0.05). EG: eggs; 1st: first instar nymphs; 2nd: second instar nymphs; 3rd: third instar nymphs; 4th: fourth instar nymphs: 5th: fifth instar nymphs; AD: adults. The mRNA level in the eggs was arbitrarily taken as 1.0.

The mRNA expression of these four *Hsps* at all developmental stages of *O*. *chinensis* is shown in Fig 3. An ANOVA showed that the relative mRNA expression of *OcGrp78* was not significantly different (*P* = 0.09) at the various stages. However, *OcHsp70*, *OcHsp90*, and *OcHsp40* mRNA expression was significantly different (*OcHsp70*, *P<*0.001; *OcHsp90*, *P* = 0.017; *OcHsp40*, *P<*0.001) at the different developmental stages. The *OcHsp70* transcript levels in 3rd and 4th instar nymphs were 11.6 and 11.5 times higher than in the eggs, respectively. Although the relative *OcHsp90* and *OcHsp40* mRNA levels differed over time, the changes in mRNA levels were similar. The mRNA levels of *OcHsp90* and *OcHsp40* increased from eggs to 4th instar nymphs but declined in 5th instar nymphs and adults.

### The effect of acute Cd on *OcHsps* mRNA expression

**Fig 4:**
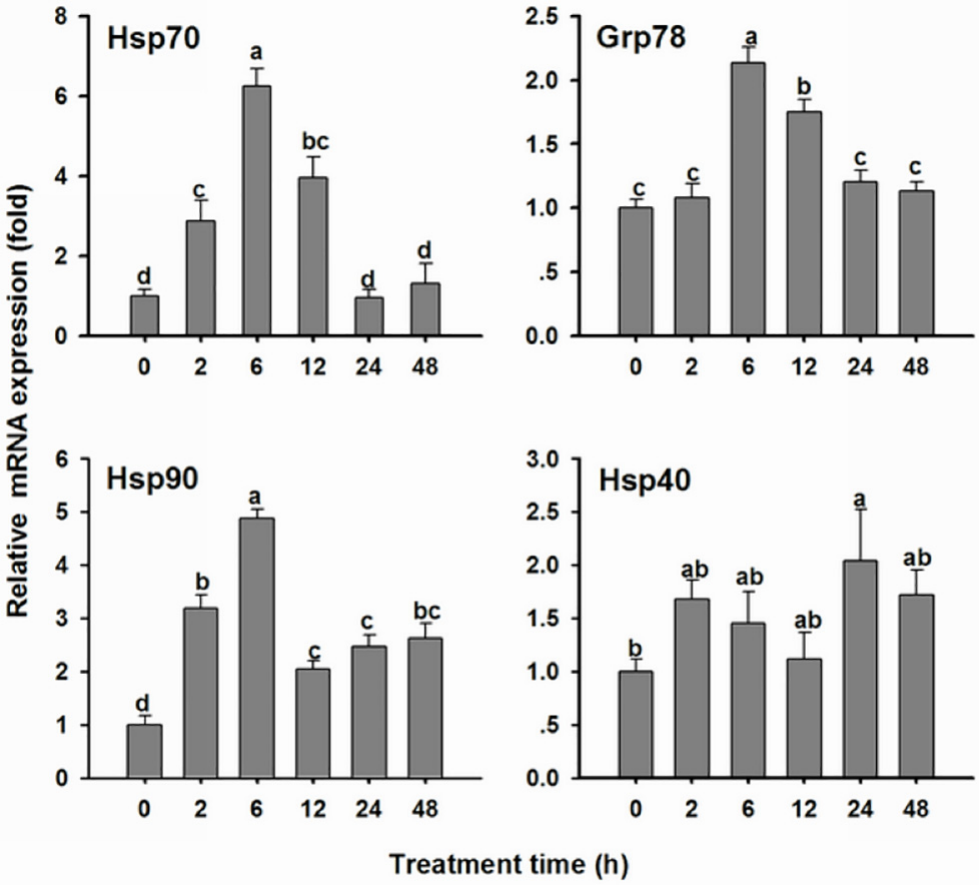
mRNA expression of *O. chinensis Hsp*s at different times after acute Cd exposure. The bars represent the mean ± SE (n = 3) of target gene mRNA expression in 5th instar nymphs 0 h, 2 h, 6 h, 12 h, 24 h and 48 h after the injection of 2.7 mg·kg^-1^ Cd. The small letters on the bars indicate significant differences between the different treatment times (Duncan’s multiple comparison, *P*< 0.05). The mRNA level at 0 h was arbitrarily taken as 1.0.

As shown in Fig 4, *OcHsp70* transcript levels began to increase 2 h after Cd treatment, reaching more than 6.2-fold those of the control at 6 h and followed by a rapid decrease at 24 h. *OcGrp78* mRNA levels also reached more than 2.1-fold those of the control at 6 h, but the decrease in *OcGrp78* levels occurred less rapidly than that for *OcHsp70*. *OcHsp90* mRNA levels increased up to 4.9-fold those of the control 6 h after treatment. At 12 h, *OcHsp90* levels decreased to approximately 2.0-fold those of the control, and these levels were maintained for 48 h. *OcHsp40* expression increased to 2.0-fold that of the control until 24 h, followed by a decrease at 48 h. These results show that *OcHsp70*, *OcGrp78*, and *OcHsp90* (but not *OcHsp40*) mRNA levels are highest after 6 h of exposure to 2.7 mg·kg^-1^ Cd.

**Fig 5:**
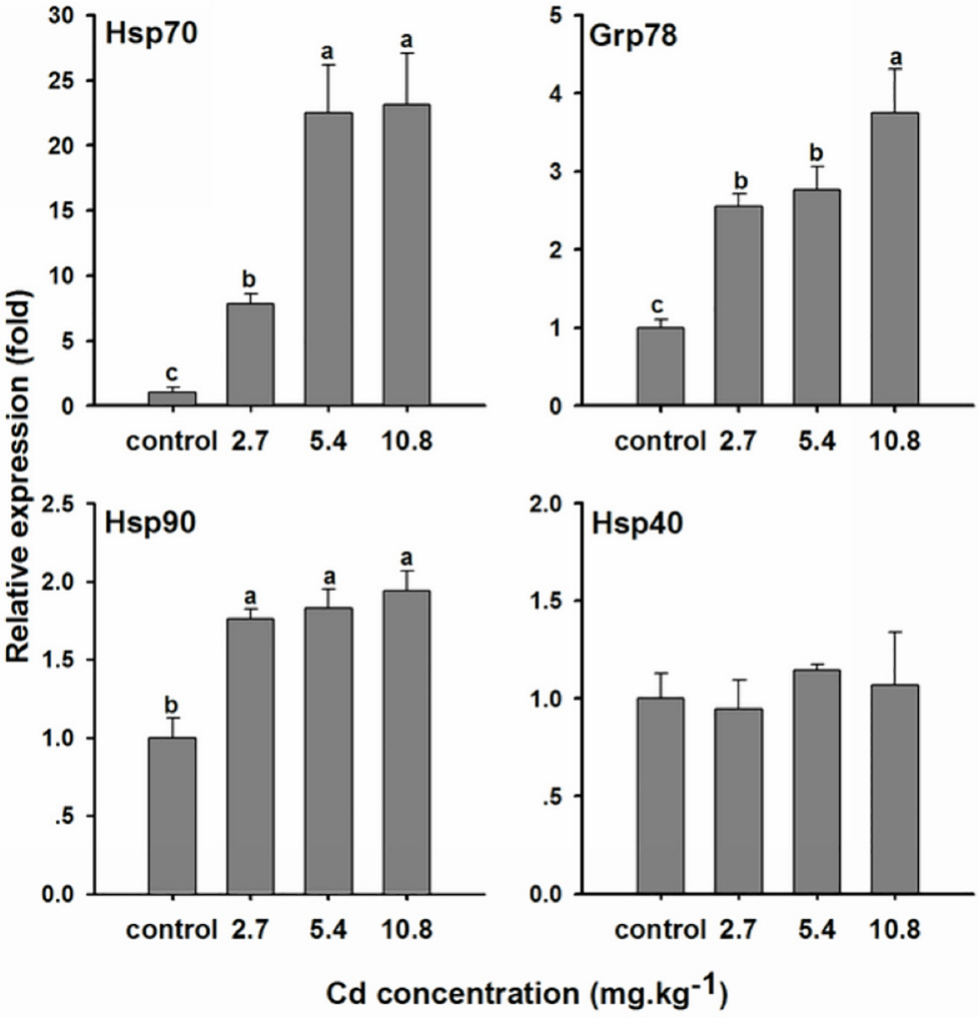
mRNA expression of *O. chinensis Hsps* 6 h after exposure to different Cd concentrations. The bars represent the mean ± SE (n = 3) of target gene mRNA expression in 5th instar nymphs 6 h after the injection of different Cd concentrations. The small letters on the bars represent significant differences between different Cd concentrations (Duncan’s multiple comparison, *P*< 0.05). Distilled water was used as a control. The value of the control was arbitrarily taken as 1.0.

The above results show that the expression of all the examined *Hsp* genes, except for *OcHsp40*, reached their highest levels at 6 h. To evaluate changes in *Hsp* gene transcript levels with different Cd concentrations, mRNA levels were analyzed by qPCR in control 5th instar nymphs and 5th instar nymphs exposed to different Cd concentrations for 6 h. As shown in Fig 5, *OcHsp70* expression was increased to 7.8-, 22.5-, and 23.1-fold that of the control after exposure to the various Cd concentrations. These results suggest that *OcHsp70* is induced in a dose-dependent manner. After a 6 h exposure, *OcGrp78* was also activated by Cd, but no difference in *OcGrp78* mRNA levels was found between the lower Cd concentration (2.7 mg·kg^-1^) and the middle Cd concentration (5.4 mg·kg^-1^). However, *OcGrp78* transcript levels were increased by 3.8-fold after exposure to the highest Cd concentration (10.8 mg·kg^-1^) compared with the control. *OcHsp90* expression increased after Cd acute exposure, but no significant differences in expression were observed with increasing Cd concentrations. No difference in *OcHsp40* expression was observed in the Cd-treated groups relative to the controls.

### The effect of chronic cadmium exposure on *OcHsps* mRNA levels

**Fig 6:**
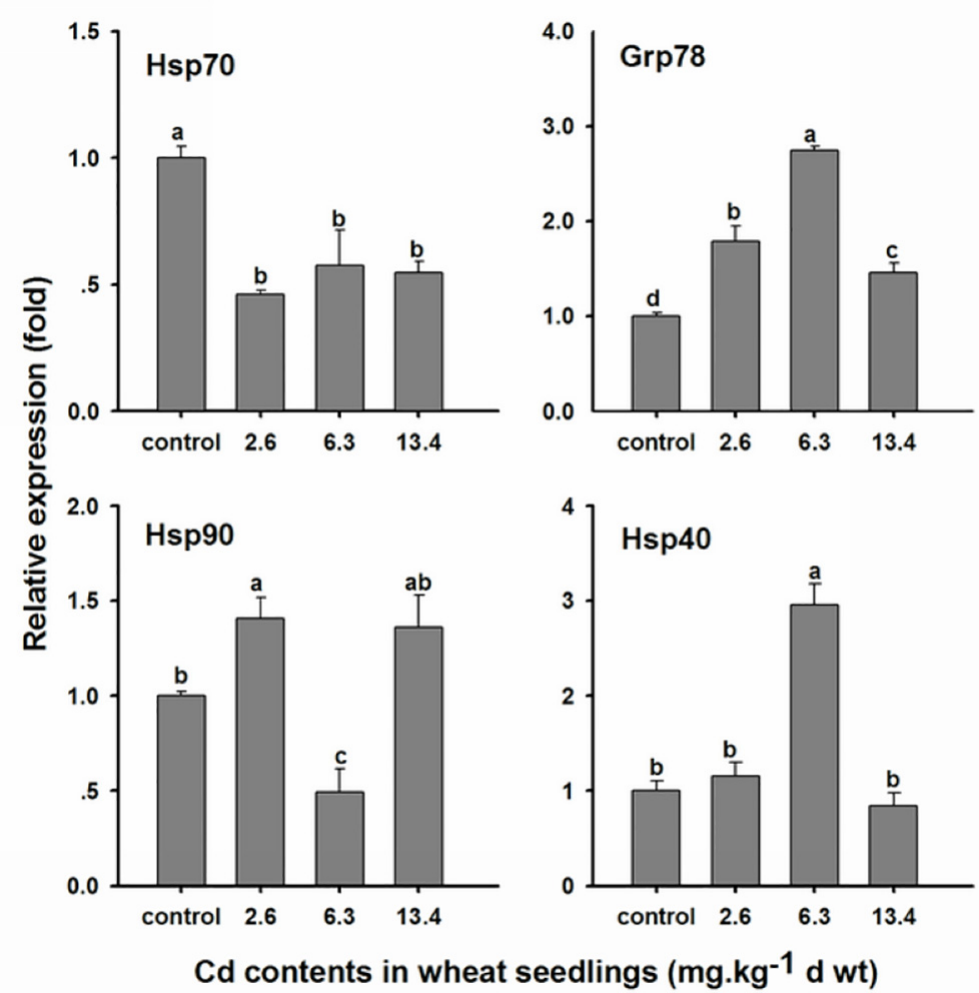
mRNA expression of *O. chinensis Hsps* after chronic Cd exposure. Wheat seedlings cultured in distilled water were used as a control. During this experiment, significant numbers of insect deaths did not occur. The bars represent the mean ± SE (n = 3) of target gene mRNA expression after chronic Cd exposure. The small letters on the bars represent significant differences between different Cd concentrations after the insects fed on dietary Cd (Duncan’s multiple comparison, *P*< 0.05). The value of the control was arbitrarily taken as 1.0.

The expression profiles of *OcHsp70*, *OcGrp78*, *OcHsp90*, and *OcHsp40* after exposure to Cd for 48 days are shown in Fig 6. In our experiments, no significant numbers of insect deaths were observed for any treatment regimen. *OcHsp70* expression significantly decreased following chronic Cd exposure compared with the control; however, no significant differences in *OcHsp70* levels were observed among the various Cd concentrations. *OcGrp78* mRNA levels first increased and then decreased with increasing Cd concentrations. *OcHsp90* expression increased at a lower Cd concentration (2.6 mg·kg^-1^) and decreased at a higher concentration (6.3 mg·kg^-1^); however, *OcHsp90* expression was equal at the highest Cd concentration (13.4 mg·kg^-1^) to that at the lowest concentration. *OcHsp40* mRNA levels increased at a Cd concentration of 6.3 mg·kg^-1^, but no significant difference was observed at the 2.6 mg·kg^-1^ and 13.4 mg·kg^-1^ Cd concentrations.

## Discussion

In this study, four *Hsp* transcripts, *Hsp70*, *Grp78*, *Hsp90*, and *Hsp40*, were cloned from *O*. *chinensis*. As expected, the typical characteristics of Hsp70 family members were observed in the deduced amino acid sequences of *OcHsp70 and OcGrp78*. The "EEVD" motif located at the C-terminus of *OcHsp70* indicates that this *Hsp70* is a cytosolic homolog. The results of previous studies investigating the "GGMP" repeat located at the C-terminus indicate that HSP70 and HSP90 might form a chaperone complex [6]. However, *OcHsp70* lacks this "GGMP" tetrapeptide repeat at the C-terminus. *OcGrp78* possesses a conserved C-terminal sequence, "KDEL", which functions as an ER localization signal. In addition, the typical motifs of Hsp70 family members were also observed in *OcHsp70*. The presence of the consensus sequence "MEEVD" indicates that *OcHsp90* is a cytosolic homolog [19]. Five Hsp90 family member domains and a conserved motif, "GXXGXG", in the *OcHsp90* amino acid sequence suggest that *OcHsp90* is functional and belongs to the Hsp90 family. The Hsp40 family has three distinct regions, the J-domain (at the N-terminal, 74 amino acids), the G/F domain, and a third motif at the C-terminal end [19–20]. These characteristic domains of Hsp40 family members are found in the N and C-termini of *OcHsp40*. The conserved "HPD" tripeptide at the DnaJ-N motif is critical for the chaperone function of Hsp40 family members [20].

Phylogenetic analysis suggests that the *OcGrp78*, *OcHsp70*, *OcHsp90*, and *OcHsp40* amino acid sequences are closest to those obtained from Orthoptera insects. In the phylogenetic tree, *OcGrp78* is closest to Grp78 in *L*. *migratoria*; this analysis also placed *Grp78* in groups containing Hsc70-3 of other insects. Five different *Hsc70* genes exist in *Drosophila melanogaster;* the encoded proteins are localized in the cytosol (Hsc70-1, Hsc70-2, and Hsc70-4), mitochondria (Hsc70-5), and endoplasmic reticulum (Hsc70-3) and have unique functions [21].OcGrp78 might be a homologous gene of Hsc70-3; this finding has not been previously reported.

We then found that the four *Hsp* mRNAs are expressed in all tissues examined, possibly because *OcGrp78*, *OcHsp70*, *OcHsp90*, and *OcHsp40* are essential for normal physiology and metabolism [22–25]. Although these four *Hsp* genes are found in all of the studied tissues, we observed higher *Hsp* mRNA levels in the fat body, Malpighian tubule, and brain. This expression pattern was similar to that of the moth *Spodoptera litura*, in which *Hsp70* and *Hsp90* expression was highest in the fat body, followed by the cuticle and midgut [26]. However, this mRNA expression pattern differs from other insects such as the flesh fly *Sarcophaga crassipalpis* [27], *Lucilia sericata* [28], and *Helicoverpa zea* [29], in which *Hsp70* and *Hsp90* expression is higher in the midgut than in other tissues. Our results suggest that the effects of metals on *OcHsp*s are not specific to digestive tissue. Indeed, the higher expression in the brain and detoxification organs (fat body and Malpighian tubule) may reflect a greater ability of these tissues to respond to Cd exposure.

The mRNA expression of the four *Hsp* genes significantly varied during all developmental stages. *OcGrp78* mRNA levels in the eggs and the 3rd instar nymphs did not significantly vary. Our results are consistent with those reported by Gonzalez-Gronow [30]. Previous studies have also illustrated that *Grp78* is essential for cell growth and pluripotent cell survival. Developmental regulation of the *Hsp70* and *Hsp90* genes in various organisms has also been reported [2, 26, 31–32]; however, only a few studies have reported on the developmental regulation of *Hsp40* in insects. In this study, *OcHsp70*, *OcHsp90* and *OcHsp40* expression significantly increased from the eggs to the 4th instar nymphs, which is consistent with findings in *L*. *migratoria* [2]. Our results are also similar to those in *Manduca sexta*, in which *Hsp70* mRNA levels tend to increase during the larval period [32]. *OcHsp70*, *OcHsp90*, and *OcHsp40* gene expression in 5th instar nymphs and adults was lower than that in 3rd and 4th instar nymphs. The expression pattern of *OcHsp70* is consistent with that found in *Drosophila*, in which pupae and adults produce less Hsp70 than do young larvae [33]. Mahroof et al. (2005) reported that a significantly greater amount (33%) of Hsp70 is synthesized in young larvae compared with that in other developmental stages in the red flour beetle *Tribolium castaneum* [31]. However, Shu et al. (2011) found that the highest *Hsp70* and *Hsp90* levels were present in adults, with the lowest observed in the 5th instar larvae of *Spodoptera litura* [26]. These differences in insect *Hsp* mRNA expression among developmental stages may be related to the physiological features of the various insects. In *O*. *chinensis*, eggs with a strong egg sac protect the insect body from stress. Therefore, eggs may have lower *Hsp* mRNA levels than the instar nymphs and adults.

In addition to metallothionein (MT), *Hsps* have been suggested as potential biomarkers of metal contamination [26]. Previous studies have indicated that acute or sub-acute Cd can induce *Hsp* expression in insects [6, 10, 26, 34–36]. We found that *OcHsp70*, *OcGrp78*, *OcHsp90* and *OcHsp40* gene expression is induced by acute Cd exposure. Among the various Hsp isoforms, Hsp70 is often the prominent protein expressed under a variety of stress conditions [22, 36]. In the present study, the *OcHsp70* gene was also the most sensitive gene under Cd acute exposure; this finding is consistent with previous reports in other insects [6, 36]. Grp78, as an ER chaperone, can be induced by Cd exposure. Organisms can increase the mRNA expression of ER chaperone proteins to improve the protein folding capacity of the ER and to resist ER stress caused by Cd exposure [37]. In our study, acute Cd exposure led to increased *OcGrp78* expression. Another possible reasons for the increase in Grp78 expression is that it participates in the protein synthesis and folding process of MT or other related proteins. In *Venerupis philippinarum*, *Hsp40* expression is first elevated and is then downregulated at 48 h in the lowest Cd concentration group [35]. In this study, the same expression pattern was observed for *OcHsp40* mRNA. In contrast, in the aquatic midge *Chironomus riparius*, *Hsp40* and *Hsp90* gene expression remained unaltered from Cd treatment [36]. However, *OcHsp90* expression was induced and reached a maximum level at 6 h. Clearly, changes in *Hsp* mRNA expression vary by magnitude, species, and duration, based on the level of Cd exposure.

Studies of the effects of chronic Cd exposure on Hsp genes are relatively few. Warchałowska-Śliwaa et al. (2005) found that the grasshopper *Tetrix tenuicornis* has decreased inducible Hsp70 levels after heavy metal exposure compared with the control [38]. Our results are consistent with this finding. We speculate that Cd accumulation in the body of insects may affect Hsp70 protein synthesis. The decrease of Hsp70 indicates that Hsp70 does not function in a protective role or alternatively, that insects exposed to chronic Cd have adapted to this environment and do not need to synthesize more Hsp70 protein [38]. *OcGrp78* expression increased at lower Cd concentrations. *Grp78* expression can be induced by intracellular Cd accumulation, and *Grp78* may participate in the unfolded protein response (UPR) signaling pathway to address Cd-induced ER stress [39]. We speculate that decreased *Grp78* expression may result from the inhibition of protein synthesis at the highest Cd concentration. Gao et al. found a clear dose-dependent *Hsp90* expression pattern in the Zhikong scallop *Chlamys farreri* after Cd exposure (50–200 μg·L^-1^) for 10–20 days [40]. *Hsp90* expression is also induced in the oyster *Crassostrea gigas* after long-term Cd exposure [34]. In our study, *OcHsp90* mRNA expression significantly increased at Cd concentrations of 2.6 mg·kg^-1^ and 13.4 mg·kg^-1^; however, *OcHsp90* expression decreased after 6.3 mg·kg^-1^ Cd exposure, which was not completely consistent with previous reports. At the concentration of 6.3 mg·kg^-1^ Cd, *OcHsp40* gene expression was induced. *Hsp40* plays an essential role in protein metabolism by regulating the polypeptide binding and release cycle of *Hsp70* [19]. The difference in expression pattern of these Oc*Hsps* may be due to a compensation effect among the *Hsp* genes. We speculate that the expression of certain *Hsp* genes is elevated to compensate for the loss of other Hsp genes under Cd stress.

## Supporting Information

S1 Fig The nucleotide and deduced amino acid sequences of *Oxya chinensis Hsp70*. The poly A tail includes one possible polyadenylation signal (AATAA) and two AU-rich elements (ARE:ATTTA). The stop codon is marked with an asterisk. Three signature sequences of the HSP70 family are shown in the blue boxes. An ATP/GTP-binding site (AEAFLGGQ) is shown in the red box. A non-organellar consensus motif (RARFEEL) is shown in the green box. The cytosolic Hsp70 motif (EEVD) of eukaryotic cells is underlined.
(DOC)

S2 Fig The nucleotide and deduced amino acid sequences of *Oxya chinensis Grp78*. The poly A tail includes one possible polyadenylation signal (AATAA) and two AU-rich elements (ARE:ATTTA). The stop codon is marked with an asterisk. Three signature sequences of the HSP70 family are shown in the blue boxes. An ATP/GTP-binding site (AEAFLGKK) is shown in the red box. A non-organellar consensus motif (RAKFEEL) is shown in the green box. An endoplasmic Hsp70 special motif (KDEL) of eukaryotic cells is underlined.
(DOC)

S3 Fig The nucleotide and deduced amino acid sequences of *Oxya chinensis Hsp90*. The poly A tail includes one possible polyadenylation signal (AATAA) and two AU-rich elements (ARE:ATTTA). The stop codon is marked with an asterisk. The five highly conserved amino acid segments that characterize all members of the Hsp90 family are shown in the blue boxes. The conserved motif (GXXGXG) is underlined in the blue boxes. The C-terminal pentapeptide MEEVD is underlined.
(DOC)

S4 Fig The nucleotide and deduced amino acid sequences of *Oxya chinensis Hsp40*. The poly A tail includes three possible polyadenylation signals (AATAA) and three AU-rich elements (ARE: ATTTA). The asterisk indicates the stop codon. Conserved DnaJ-N domain and DnaJ-C motifs are shown in the red and blue boxes, respectively. The HPD tripeptide is underlined in the red box. The G/F domain is shown in the green box.
(DOC)
