# Supplementary material for: Molecular Cloning and mRNA Expression of Heat Shock Protein Genes and Their Response to Cadmium Stress in the Grasshopper Oxya chinensis
Source: PLoS One. 2015 Jul 2;10(7):e0131244. doi: 10.1371/journal.pone.0131244 (PMC4489864; doi:10.1371/journal.pone.0131244)
Supplement: S2 Fig — The poly A tail includes one possible polyadenylation signal (AATAA) and two AU-rich elements (ARE:ATTTA). The stop codon is marked with an asterisk. Three signature sequences of the HSP70 family are shown in the blue boxes. An ATP/GTP-binding site (AEAFLGKK) is shown in the red box. A non-organellar consensus motif (RAKFEEL) is shown in the green box. An endoplasmic Hsp70 special motif (KDEL) of eukaryotic cells is underlined. (DOC) [file pone.0131244.s002.doc]

**S2 Fig. The nucleotide and deduced amino acid sequence of *Oxya chinensis Grp78.*** The poly A tail includes one possible polyadenylation signal (AATAA), and two AU-rich elements (ARE: ATTTA). The stop codon is marked with an asterisk. Three signature sequences of HSP70 family are shown in the blue boxes. An ATP/GTP-binding site (AEAFLGKK) is shown in the red box. A non-organellar consensus motif (RAKFEEL) is shown in the green box. An endoplasmic Hsp70 special motif (KDEL) of eukaryotic cells is underlined.
