# Supplementary material for: Molecular Cloning and mRNA Expression of Heat Shock Protein Genes and Their Response to Cadmium Stress in the Grasshopper Oxya chinensis
Source: PLoS One. 2015 Jul 2;10(7):e0131244. doi: 10.1371/journal.pone.0131244 (PMC4489864; doi:10.1371/journal.pone.0131244)
Supplement: S3 Fig — The poly A tail includes one possible polyadenylation signal (AATAA) and two AU-rich elements (ARE:ATTTA). The stop codon is marked with an asterisk. The five highly conserved amino acid segments that characterize all members of the Hsp90 family are shown in the blue boxes. The conserved motif (GXXGXG) is underlined in the blue boxes. The C-terminal pentapeptide MEEVD is underlined. (DOC) [file pone.0131244.s003.doc]

**S3 Fig. The nucleotide and deduced amino acid sequence of *Oxya chinensis Hsp90*.** The poly A tail includes one possible polyadenylation signal (AATAA), and two AU-rich elements (ARE: ATTTA). The stop codon is marked with an asterisk. The five highly conserved amino acid segments that characterize all members of Hsp90 family are shown in the blue boxes. The conserved motif (GXXGXG) is underlined in the blue boxes. The C-terminal pentapeptide MEEVD is underlined.
