# Supplementary material for: Molecular Cloning and mRNA Expression of Heat Shock Protein Genes and Their Response to Cadmium Stress in the Grasshopper Oxya chinensis
Source: PLoS One. 2015 Jul 2;10(7):e0131244. doi: 10.1371/journal.pone.0131244 (PMC4489864; doi:10.1371/journal.pone.0131244)
Supplement: S4 Fig — The poly A tail includes three possible polyadenylation signals (AATAA) and three AU-rich elements (ARE: ATTTA). The asterisk indicates the stop codon. Conserved DnaJ-N domain and DnaJ-C motifs are shown in the red and blue boxes, respectively. The HPD tripeptide is underlined in the red box. The G/F domain is shown in the green box. (DOC) [file pone.0131244.s004.doc]

**S4 Fig. The nucleotide and deduced amino acid sequence of *Oxya chinensis Hsp40*.** The poly A tail includes three possible polyadenylation signal (AATAA), and three AU-rich elements (ARE: ATTTA). The asterisk indicates the stop codon. Conserved DnaJ-N domain and DnaJ-C motifs are shown in the red and blue box, respectively. The HPD tripeptide is underlined in the red box. The G/F domain is shown in the green box.
